# Implantation and tracing of green fluorescent protein-expressing adipose-derived stem cells in peri-implant capsular fibrosis

**DOI:** 10.1186/s13287-023-03248-0

**Published:** 2023-02-08

**Authors:** Bo-Yoon Park, Dirong Wu, Kyoo-Ri Kwon, Mi-Jin Kim, Tae-Gon Kim, Jun-Ho Lee, Do Young Park, Il-Kug Kim

**Affiliations:** 1 Department of Plastic and Reconstructive Surgery, Yeungnam University College of Medicine, 170, Hyeonchung-ro, Nam-gu, Daegu, 42415 Korea; 2 Department of Ophthalmology, Yeungnam University College of Medicine, 170, Hyeonchung-ro, Nam-gu, Daegu, 42415 Korea

**Keywords:** Breast reconstruction, Breast implant, Capsular contracture, Stem cells, Cell differentiation

## Abstract

**Background:**

Adipose-derived stem cells (ASCs) have been reported to reduce fibrosis in various tissues. In this study, we investigated the inhibitory role of ASCs on capsule formation by analyzing the histologic, cellular, and molecular changes in a mouse model of peri-implant fibrosis. We also investigated the fate and distribution of ASCs in the peri-implant capsule.

**Methods:**

To establish a peri-implant fibrosis model, customized silicone implants were inserted into the dorsal site of C57BL/6 wild-type mice. ASCs were harvested from the fat tissues of transgenic mice that express a green fluorescent protein (GFP-ASCs) and then injected into the peri-implant space of recipient mice. The peri-implant tissues were harvested from postoperative week 2 to 8. We measured the capsule thickness, distribution, and differentiation of GFP-ASCs, as well as the cellular and molecular changes in capsular tissue following ASC treatment.

**Results:**

Injected GFP-ASCs were distributed within the peri-implant capsule and proliferated. Administration of ASCs reduced the capsule thickness, decreased the number of myofibroblasts and macrophages in the capsule, and decreased the mRNA level of fibrogenic genes within the peri-implant tissue. Angiogenesis was enhanced due to trans-differentiation of ASCs into vascular endothelial cells, and tissue hypoxia was relieved upon ASC treatment.

**Conclusions:**

We uncovered that implanted ASCs inhibit capsule formation around the implant by characterizing a series of biological alterations upon ASC treatment and the fate of injected ASCs. These findings highlight the value of ASCs for future clinical applications in the prevention of capsular contracture after implant-based reconstruction surgery.

## Background

Capsular contracture is a major complication that occurs after implant-based breast reconstruction or augmentation, leading to excessively firm and painful breasts [1–3]. Although surgical treatments such as capsulotomy and capsulectomy are generally successful, failure and relapse are common occurrences [4, 5]. Capsular contracture is a type of reaction to a foreign body. These capsules typically represent dense fibrous connective tissue containing foreign giant cells or granulomas, suggesting that they are caused by inflammatory responses [6]. Systemic factors related to the wound healing process and various local factors that stimulate inflammation are both considered to induce massive collagen production by fibroblasts and myofibroblasts, leading to excessive fibrosis. However, the exact molecular mechanisms through which these processes occur remain unclear [7, 8].

Many studies examining the mechanism of capsular contracture have employed silicone implantation into animal models [9–12]. Factors such as tissue hypoxia, the presence of bacteria, the texture of the implant, and transforming growth factor β (TGFβ) signaling have all been linked to the occurrence of capsular contracture [10, 11, 13, 14]. Acellular dermal matrices and anti-adhesive agents were recently reported to reduce capsular contracture by inhibiting myofibroblasts and macrophages [7, 15]. Therefore, identifying novel alternatives for minimizing capsular contracture is crucial to further our understanding of the molecular mechanisms of capsular contracture and for the establishment of new therapeutics.

Adipose-derived stem cells (ASCs) have been suggested as a treatment option for fibrosis in the lung, liver, heart, kidney, and other organs, due to their anti-fibrotic, immunomodulatory, and angiogenic effects [16–18]. We hypothesized that ASCs could reduce the capsular contracture around the implant. In this study, we used a silicone-implanted mouse capsular fibrosis model to investigate the role of ASCs in capsular contracture and the underlying mechanisms. In particular, we used ASCs harvested from transgenic mice that produce a green fluorescent protein (GFP) in all of their cells to track the implantation and differentiation of ASCs around the implant [19]. In addition, we examined the histopathologic features and molecular changes in peri-implant capsules upon ASC supplementation.

## Methods

### Animal care and surgical implantation

Wild-type C57BL/6 (B6) and GFP-B6 mice (8–12 weeks old and 20–25 g) were bred in specific pathogen-free animal facilities at Yeungnam University College of Medicine, Daegu, Korea. GFP-B6 mice were kindly provided by professor Hak Chang (Seoul National University, Korea) [19]. Animal care and experimental procedures were approved by the Institutional Animal Care and Use Committee of Yeungnam University College of Medicine (No. YUMC-2020–037; Title: Therapeutic effect of ASC injection in a mouse model of capsular formation around implants; Date of approval: December 1, 2020). Six mice were allocated to each group, and a total of 54 wild-type mice were used in this study. The sample size was determined by previous literatures [7, 19, 20]. There was no excluded mice from the study. Customized silicone implants with a smooth surface (2 cm in diameter, 1 mL in volume, BNS Med, Seoul, Korea) were implanted into B6 wild-type mice. Mice were anesthetized through intraperitoneal injection of a combination of ketamine (100 mg/mL) and rumpun (12.5 mg/mL). The surgical site was shaved and prepared with an iodine solution. A 2.5-cm longitudinal incision was made on the dorsal side of the mouse. Next, a pocket was dissected into the subcutaneous plane and the customized silicone implants were placed between the muscle and subcutaneous layer. The incision was then closed with interrupted sutures using 4-0 silk (Ailee Co. Ltd. Busan, Korea). If the silicone implant was exposed caused by inflammation, it was decided to exclude the mouse from the experiment. After capsule tissue harvest, mice were humanely euthanized by CO_2_ asphyxiation followed by cervical dislocation.

### Culture and administration of ASCs from GFP-positive mice

Subcutaneous and visceral fat harvested from 9 GFP-B6 mice were digested in 0.2% collagenase type I (Worthington Biochemical Corporation, Lakewood, USA) for 20 min at 37 °C with continuous agitation. After the inactivation of collagenase activity, suspended cells were filtered through 100-μm and 40-μm cell strainers and centrifuged for 5 min at 1,000 rpm. After removing the floating adipocytes and supernatant, the pellets were resuspended in culture media containing DMEM/High glucose (HyClone, Logan, USA), 10% fetal bovine serum, and 1% penicillin/streptomycin and incubated in 5% CO_2_ at 37 °C. Cultured ASCs were confirmed to express stem cell markers such as CD90, CD105, and CD73 by flow cytometry and have a capacity for multipotent differentiation into adipocytes, osteocytes, and chondrocytes without an endothelial or hematopoietic lineage-positive subpopulation [19, 20]. One million GFP-expressing ASCs (GFP-ASCs) suspended in 100 μL phosphate-buffered saline (PBS) were injected into the peri-implant space of recipient mice with a 24-gauge needle at postoperative week 1. Peri-implant capsules were harvested from postoperative week 2 to 8.

### Histologic analysis

Harvested capsules were fixed in 4% paraformaldehyde diluted in PBS overnight. For hematoxylin and eosin (H&E) staining, capsule tissues were embedded in paraffin after fixation. The stained slides were scanned using a nanoscope system. For immunofluorescent staining, the capsules were sequentially dehydrated in 20% and 30% sucrose solutions after fixation, then cryoembedded and cryosectioned. The cryosectioned slides were blocked with 5% bovine serum albumin diluted in PBS for 1 h at 37 °C, and then incubated with one of the following primary antibodies overnight at 4 °C: rabbit anti-mouse GFP antibody (AB3080; Millipore, Darmstadt, Germany), goat anti-mouse GFP antibody (ab5450; Abcam, Cambridge, UK), rat anti-mouse Ki67 antibody (14-5698-82; Invitrogen, Waltham, USA), rabbit anti-mouse PH3 antibody (9664; Cell signaling, Danvers, USA), rabbit anti-mouse Caspase3 antibody (53348; Cell signaling, Danvers, USA), mouse Cy3-conjugated anti-α-smooth muscle actin (αSMA) antibody (clone 1A4; Merck, Kenilworth, USA), rat anti-mouse F4/80 antibody (MAB5580; R&D Systems, Minneapolis, USA), mouse anti-mouse inducible nitric oxide synthase (iNOS) antibody (MAB9502; R&D Systems, Minneapolis, USA), goat anti-mouse CD206 antibody (AF2535; R&D Systems, Minneapolis, USA), or an Armenian hamster anti-mouse CD31 antibody (clone 2H8; Millipore, Darmstadt, Germany). On day two, the tissues were washed two times and then incubated with one of the following secondary antibodies: a goat Alexa488- or Alexa647-conjugated anti-rabbit IgG antibody (Jackson ImmunoResearch, West Grove, USA), an Alexa647-conjugated anti-Rat IgG antibody (Jackson ImmunoResearch), an Alexa594- or Alexa647-conjugated anti-mouse IgG antibody (Jackson ImmunoResearch), an Alexa488- or Alexa594- or Alexa647-conjugated anti-goat IgG antibody (Jackson ImmunoResearch), or an Alexa647-conjugated anti-Armenian Hamster IgG antibody (Jackson ImmunoResearch) for 2 h at room temperature. DAPI (4′,6-diamidino-2-phenylindole was used as a nuclear counterstain, and VECTASHIELD® PLUS Antifade Mounting Medium (H-2000; Vector Lab, Burlingame, USA) was used to mount coverslips. A confocal laser scanning microscope (LSM 800; Zeiss, Oberkochen, Germany) was used to capture fluorescent images.

The percentage of GFP-positive ASCs was defined by examining the number of GFP-positive areas per random 1.638 mm^2^ regions. The percentage of Ki67, PH3, Caspase3, αSMA, and F4/80-positive cells was measured as percentages of the corresponding fluorescent-positive areas per random 0.408 mm^2^ regions. iNOS and F4/80 co-positive M1 macrophage polarity was defined by the percentage of iNOS-positive areas divided by F4/80-positive areas per random 0.408 mm^2^ regions. CD206 and F4/80 co-positive M2 macrophage polarity was defined as the percentage of CD206-positive areas divided by F4/80-positive areas per random 0.408 mm^2^ regions. The vessel area was assessed as the number of CD31-positive areas per random 0.408 mm^2^ regions. Immunofluorescence signal densities were quantified using the ImageJ software (National Institutes of Health, Bethesda, USA) in a blind manner.

### Hypoxia assessment

To analyze the hypoxic areas of capsule tissue, 60 mg/kg pimonidazole hydrochloride (HP-ATTO 594, Natural Pharmacia International, Burlington, USA) was intraperitoneally administered 60 min before the capsule tissues were harvested [21–23]. Mice were anesthetized and intracardially perfused with PBS to eliminate circulating pimonidazole. Capsule tissues were stained with an ATTO 594 fluorophore-conjugated anti-hypoxyprobe antibody. Hypoxic areas were assessed as percentages of the ATTO 594 fluorophore-positive area per random 1.638 mm^2^ regions. All signal intensities were measured using the ImageJ software.

### Gene expression analysis

Isolated tissues were immediately frozen using liquid nitrogen and homogenized. Total RNA was isolated using the QIAzol lysis reagent (Qiagen, Hilden, Germany), and reverse transcription was performed using a RevertAid First Strand cDNA Synthesis Kit (Thermo Fisher Scientific, Waltham, USA) according to the manufacturers’ instructions. Quantitative real-time polymerase chain reaction (qRT-PCR) was performed using the CFX Connect real-time PCR detection system (BIO-RAD, Hercules, USA). We examined the expression of hypoxia/inflammation markers (Hypoxia-inducible factor 1α [*Hif1α*], *Tgfβ*, interleukin 1-beta [*Il-1β*], interleukin 6 [*Il-6*]) and fibrogenesis markers (*Fibronectin* [*Fn*], *Collagen type I alpha 1* [*Col1α1*], and *Collagen type III alpha 1* [*Col3α1*]) in both groups. Relative changes in gene expression were calculated using the ΔCt method with normalization to a housekeeping gene (Glyceraldehyde 3-phosphate dehydrogenase [GADPH]). The sequences of primers used in this study are listed in Table 1.

**Table 1:** Primer sequences used for qRT-PCR

Gene	Forward (5′-3′)	Reverse (5′-3′)
GADPH	CATCACTGCCACCCAGAAGACTG	ATGCCAGTGAGCTTCCCGTTCAG
TGF-β	CTGCTGACCCCCACTGATAC	AGCCCTGTATTCCGTCTCCT
Hif-1α	CCTGCACTGAATCAAGAGGTTGC	CCATCAGAAGGACTTGCTGGCT
Interleukin-1β	TGGACCTTCCAGGATGAGGACA	GTTCATCTCGGAGCCTGTAGTG
Interleukin-6	TGGTTCCTACCCCAATTTCCA	GTCTTGGTCCTTAGCCACTCC
Collagen type 1α1	CCTCAGGGTATTGCTGGACAAC	CAGAAGGACCTTGTTTGCCAGG
Collagen type 3α1	GACCAAAAGGTGATGCTGGACAG	CAAGACCTCGTGCTCCAGTTAG
Fibronectin	CCCTATCTCTGATACCGTTGTCC	TGCCGCAACTACTGTGATTCGG

### Statistical analysis

All data were analyzed using independent *t*-tests to compare two groups or one-way analysis of variance to compare three groups. Data are presented as means (M) ± standard deviations (SD). Differences with *p*-values < 0.05 were considered statistically significant. Statistical analyses were performed using the SPSS statistical software (version 22, IBM, Armonk, USA).

## Results

### GFP-ASC injection reduced peri-implant capsule thickness

**Fig. 1:**
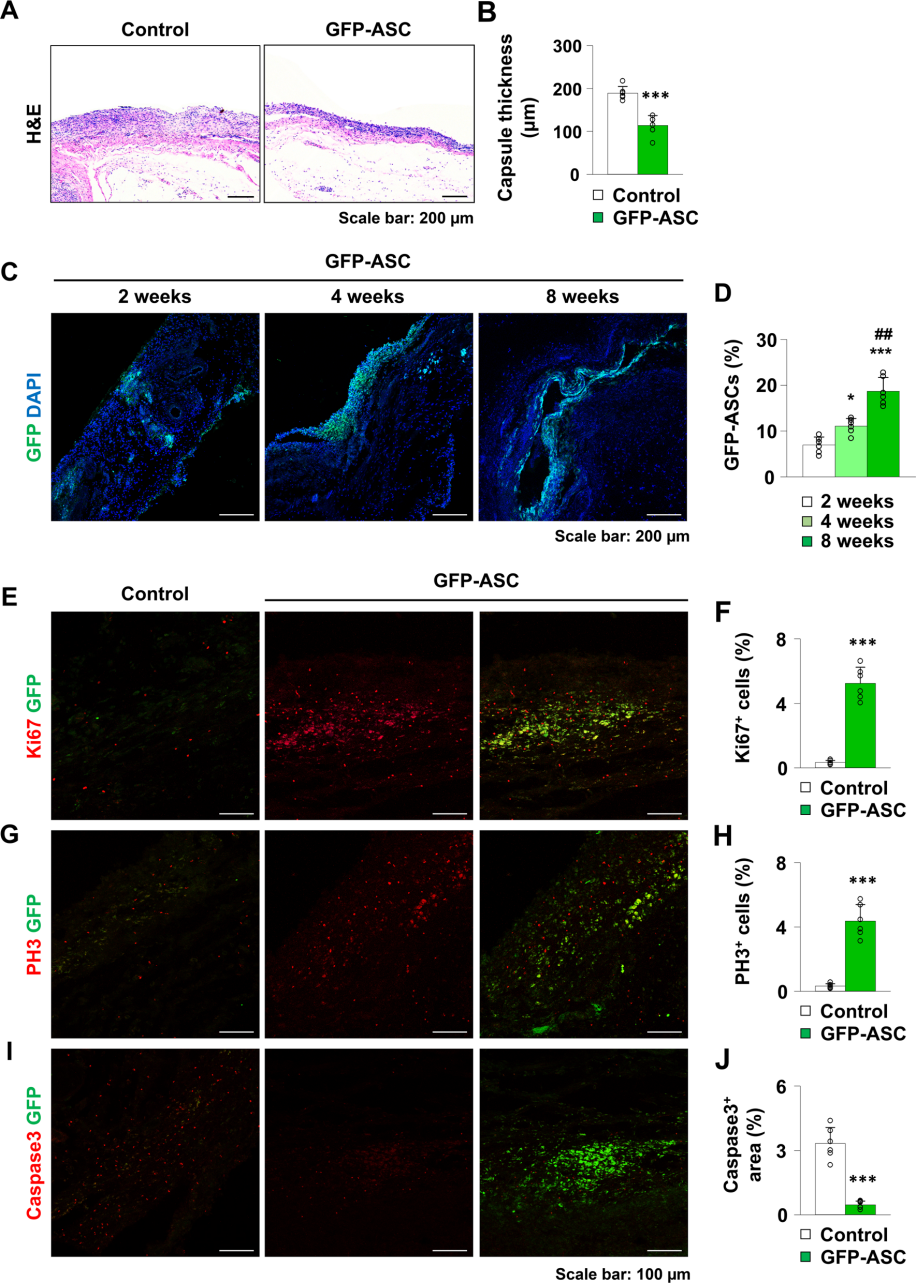
**A**, **B** Representative images and quantification of the peri-implant capsule thickness in the control and green fluorescent protein-adipose-derived stem cell (GFP-ASC) groups. **C**, **D** Immunofluorescence staining showing the serial distribution of GFP-ASCs injected into the peri-implant space from postoperative week 2 to 8. **E**, **F** Representative images and quantification of Ki67-positive cells in the control and GFP-ASC groups. **G**, **H** Representative images and quantification of PH3-positive cells in the control and GFP-ASC groups. **I**, **J** Representative images and quantification of Caspase3-positive areas in the control and GFP-ASC groups. Values are presented as mean ± SD (*n* = 6). Scale bars = 200 μm (**A**, **C**); 100 μm (**E**, **G**, **I**). *p < 0.05, ***p < 0.001 versus control or 2 weeks. ## *p* < 0.01 versus 4 weeks. DAPI, 4′,6-diamidino-2-phenylindole

First, we investigated whether injection of GFP ASCs could reduce peri-implant capsule thickness. Peri-implant capsules were harvested from all control and GFP-ASC-treated mice at postoperative week 8. The peri-implant capsule was significantly thinner in the GFP-ASC group than that in the control group, which suggests that ASC treatment reduces capsular fibrosis (control group: M = 189.21 ± SD = 15.77 μm; GFP-ASC group: M = 113.96 ± SD = 23.18 μm; *p* < 0.001; Fig. 1A, B).

### Distribution of ASCs in the peri-implant capsule

To confirm that injected ASCs are viable and properly distributed in the fibrous capsule adjacent to the silicone implant, we examined GFP fluorescence in the mice in the ASC group 2, 4, and 8 weeks after ASC injection. ASCs expressing GFP were mainly distributed in the fibrous tissues that closely contact the silicone implant, confirming their viability at every time point (Fig. 1C). We also found that the distribution of GFP-ASCs gradually increased from 2 to 8 weeks after injection, suggesting that injected GFP-ASCs may undergo significant proliferation (Fig. 1C, D).

To analyze whether the increase in GFP-ASCs in the peri-implant tissue (from 2 to 8 weeks) following GFP-ASC injection was due to an increase in proliferation or a decrease in apoptosis, the expression of proliferation markers Ki67 and PH3 and the apoptosis marker Caspase3 was investigated. As a result, a significant proliferation without apoptosis was observed in GFP-expressing ASCs in the peri-implant tissue (Fig. 1E–J), which may contribute to the gradual increase in GFP-ASCs around the implant after injection ([Ki67, control group: M = 0.33 ± SD = 0.13%; GFP-ASC group: M = 5.25 ± SD = 1.00%; *p* < 0.001; Fig. 1E, F] [PH3, control group: M = 0.32 ± SD = 0.14%; GFP-ASC group: M = 4.37 ± SD = 1.02%; *p* < 0.001; Fig. 1G, H] [Caspase3, control group: M = 3.32 ± SD = 0.75%; GFP-ASC group: M = 0.46 ± SD = 0.17%; *p* < 0.001; Fig. 1I, J]).

### ASC injection decreased myofibroblast density in the peri-implant tissue

**Fig. 2:**
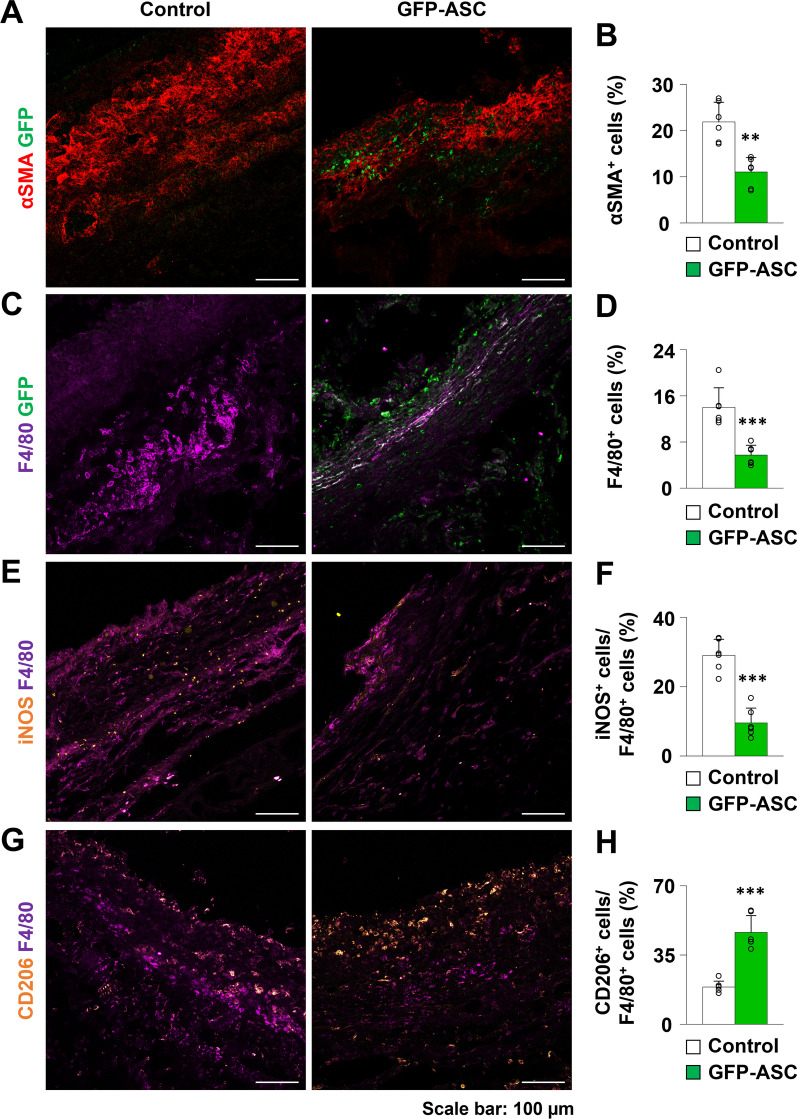
**A**, **B** Representative images and quantification of α-smooth muscle actin (αSMA) in the control and green fluorescent protein-adipose-derived stem cell (GFP-ASC) groups. **C**, **D** Representative images and quantification of F4/80-positive macrophages in the control and GFP-ASC groups. **E**, **F** Representative images and quantification of iNOS-positive cells in F4/80-positive macrophages in the control and GFP-ASC groups. **G**, **H** Representative images and quantification of CD206-positive cells in F4/80-positive macrophages in the control and GFP-ASC groups. Values are presented as mean ± SD (*n* = 6). Scale bars = 100 μm. **p < 0.01. ***p < 0.001

Our finding that the peri-implant capsule became thinner after GFP-ASC injection led us to investigate the density of myofibroblasts, which produce an excessive extracellular matrix that leads to tissue fibrosis [24]. We investigated the expression of a myofibroblast marker, αSMA, in the peri-implant capsule. The density of αSMA-positive myofibroblasts was lower in the GFP-ASC group than that in the control group (control group: M = 21.89 ± SD = 4.28%; GFP-ASC group: M = 11.02 ± SD = 3.18%; *p* = 0.001; Fig. 2A, B). These data suggest that GFP-ASCs grafted in peri-implant tissues may reduce capsule formation by decreasing myofibroblast density.

### GFP-ASC injection decreased macrophage density in peri-implant tissue

Macrophages are recruited and activated during the inflammatory process and lead to fibrosis by activating myofibroblasts [25]. To evaluate the degree of inflammation in the peri-implant capsular tissue, we quantified macrophage infiltration by measuring the density of F4/80-expressing macrophages. The density of F4/80-positive macrophages in capsular tissue was significantly lower in the GFP-ASC group than that in the control group, suggesting that inflammation was relieved by GFP-ASC injection of peri-implant capsules (control group: M = 14.02 ± SD = 3.38; GFP-ASC group: M = 5.73 ± SD = 1.71; *p* < 0.001; Fig. 2C, D). Further characterization of the polarity of M1/M2 macrophages by immunofluorescence analysis revealed that proportion of the pro-inflammatory M1 macrophages to the anti-inflammatory M2 macrophages was lower in the GFP-ASC group, which supports the anti-inflammatory effect of the ASCs ([iNOS/F4/80, control group: M = 29.06 ± SD = 4.60%; GFP-ASC group: M = 9.56 ± SD = 4.27%; *p* < 0.001; Fig. 2E, F] [CD206/F4/80, control group: M = 18.82 ± SD = 3.02%; GFP-ASC group: M = 46.44 ± SD = 8.50%; *p* < 0.001; Fig. 2G, H]).

### Differentiation of GFP-ASCs into vascular endothelial cells enhances angiogenesis in the peri-implant capsule

**Fig. 3:**
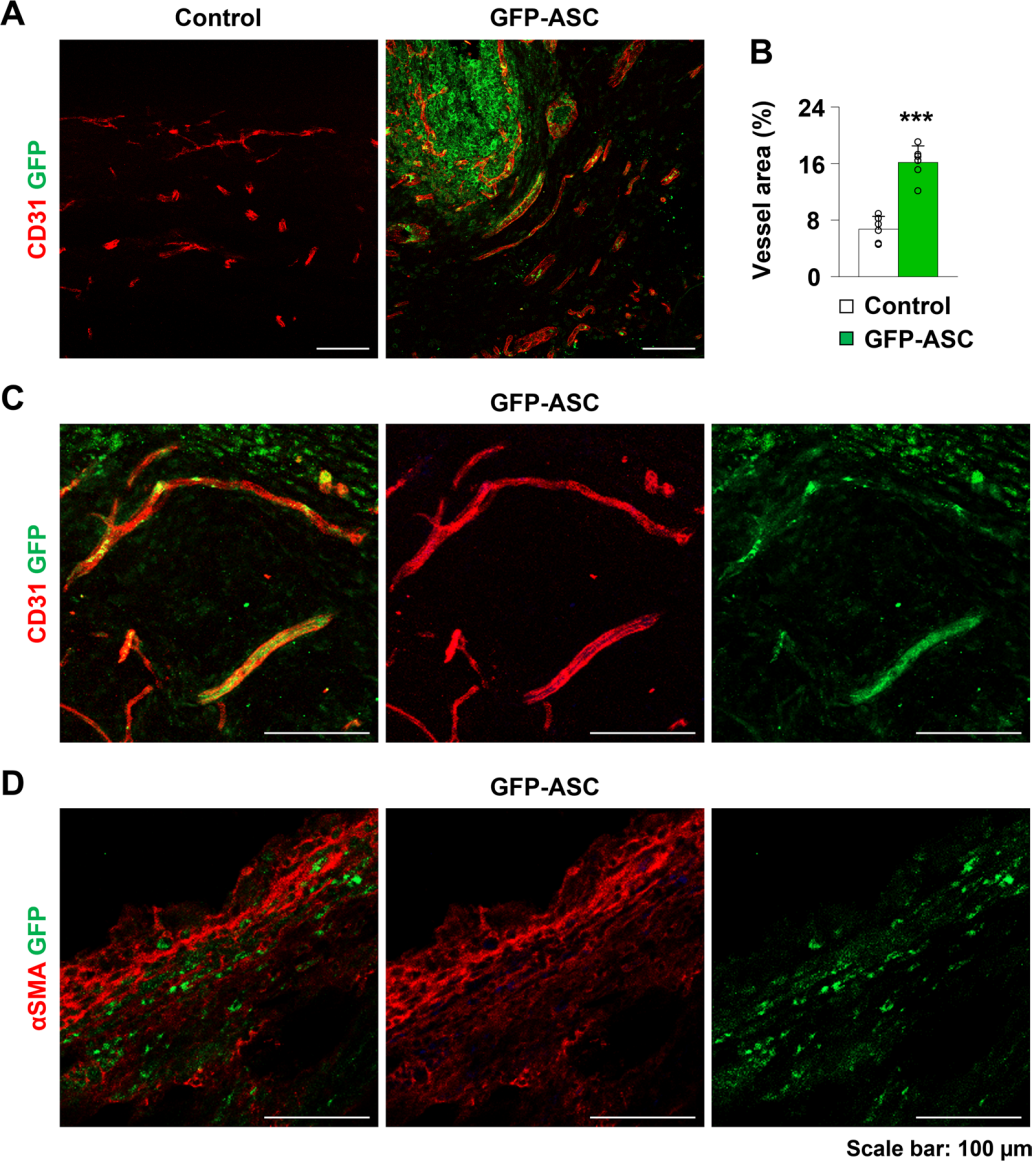
**A**, **B** Representative images and quantification of CD31 in the control and green fluorescent protein-adipose-derived stem cell (GFP-ASC) groups. **C** Representative immunofluorescent images of vessels that are co-positive for CD31 and GFP in the peri-implant capsule. **D** Representative immunofluorescent images of αSMA and GFP in peri-implant capsule. Values are presented as mean ± SD (*n* = 6). Scale bars = 100 μm. ***p < 0.001

Fibrosis is known to be linked to the vascularity and hypoxic status of the tissue [26]. To investigate the vascularity of peri-implant capsules in the control and ASC groups, we analyzed the expression of CD31 by immunofluorescence. Vascular density in the ASC group was higher than that in the control group (control group: M = 6.71 ± SD = 1.81; GFP-ASC group: M = 16.15 ± SD = 2.35; *p* < 0.001; Fig. 3A, B). Next, to determine the fate of the injected ASCs within the capsular tissue, we evaluated GFP signals in the CD31-expressing vascular endothelial cells and αSMA-expressing myofibroblasts. αSMA-positive myofibroblasts did not overlap with GFP-ASCs in the peri-implant capsule (Fig. 3D). Meanwhile, vascular endothelial cells co-stained for GFP and CD31 were observed in the peri-implant capsule tissue (Fig. 3B). These results indicate that the differentiation of injected GFP-ASCs into vascular endothelial cells can enhance angiogenesis within the peri-implant capsule.

### ASC treatment altered the levels of fibrogenic genes and relieved hypoxia

**Fig. 4:**
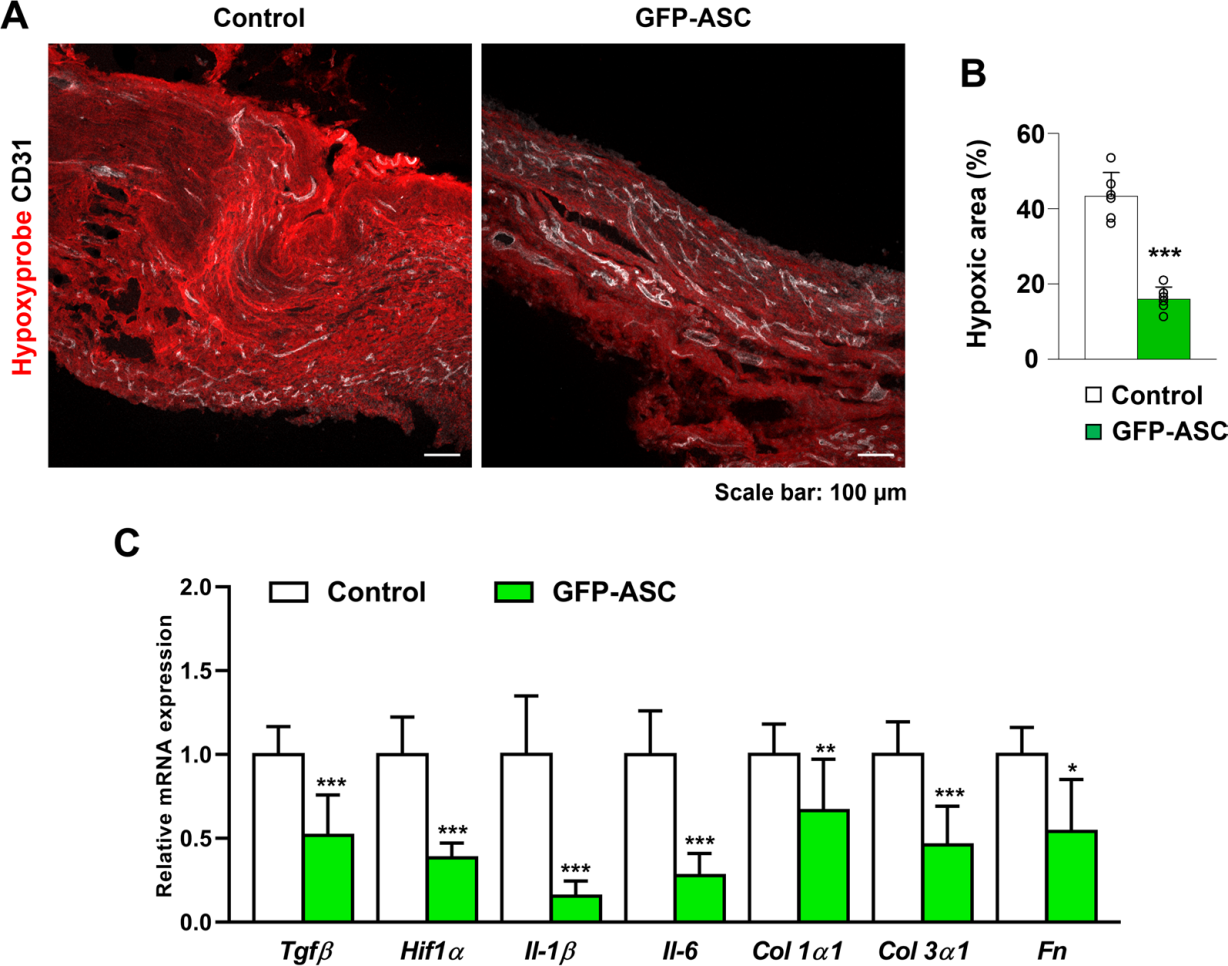
**A**, **B** Representative images and quantification of hypoxyprobe and CD31 in the control and green fluorescent protein-adipose-derived stem cell (GFP-ASC) groups. The percentage of the peri-implant capsule that is hypoxic is lower in the GFP-ASC group than in the control group. **C** Gene expression analysis examining *Tgfβ*, *Hif1α*, *Il-1β*, *Il-6, Col1α1*, *Col3α1*, and *Fn* levels using quantitative real-time polymerase chain reaction in the control group and green fluorescent protein-adipose-derived stem cell (GFP-ASC) group. GAPDH was used as a housekeeping gene for normalization. Values are presented as mean ± SD (*n* = 6). Scale bars = 100 μm. *p < 0.05, **p < 0.01, and ***p < 0.001

Excessive deposition of extracellular matrix and extensive microangiopathy induce the formation of excessive fibrous capsules and vascular rarefaction, which lead to tissue hypoxia [27]. Conversely, prolonged exposure to hypoxia leads to HIF1α upregulation, which induces TGFβ signaling and fibrosis [28–30]. Based on this, we first evaluated how GFP-ASCs affect hypoxia and fibrosis in peri-implant tissues by quantifying the hypoxic area within the peri-implant tissues and investigating their mRNA levels of HIF1α and TGFβ. The extent of the hypoxic area (control group: M = 43.29 ± SD = 6.33; GFP-ASC group: M = 15.94 ± SD = 3.25; *p* < 0.001; Fig. 4) and the levels of *HIF1α* and *TGFβ* mRNAs were significantly lower in the ASC group than those in the control group (Fig. 4C).

Histologically, fibrous tissue is characterized by excessive production and deposition of extracellular matrix components such as fibronectin and collagen, and its formation is triggered by inflammation [31–33]. Hence, we next investigated the transcript levels of genes related to fibrosis and inflammation*.* In the ASC group, the levels of *Col1α1*, *Col3α1*, *Fibronectin*, *Il-1β*, and *Il-6* mRNAs were significantly lower than those in the control group (Fig. 4C). Collectively, these findings suggest that ASCs effectively prevent capsule fibrosis by changing the microenvironment of the capsular tissues surrounding the implant, rendering it less hypoxic and less fibrogenic.

## Discussion

The prevention and treatment of capsular contracture are the major issues that are associated with managing complications after implant-based breast reconstruction and augmentation [1, 3, 5, 8]. Post-implantation tissue fibrosis is associated with a variety of factors, ranging from the type of prosthetic material used, bacterial infection, inflammatory response-mediated factors, and even surgical incision [8]. To date, several studies have sought to develop strategies to prevent and treat capsular contracture, including surgery, acellular dermal matrix [7, 34], fat grafting [35], and medication [36, 37]. In this study, we focused on cell therapy and investigated the role of ASCs in the process of capsular formation in a mouse model.

We demonstrated that GFP-ASC supplementation significantly reduced the degree of capsular fibrosis surrounding silicone implants. When GFP-ASCs were injected around a silicone implant, the thickness of the fibrous capsule, myofibroblast formation, and inflammatory cell infiltration were all decreased. Peri-implant tissue hypoxia was also relieved and angiogenesis was increased as a result of ASCs differentiating into vascular endothelial cells. In addition, we found that the synthesis of TGFβ and extracellular matrix molecules that induce fibrosis was decreased upon ASC treatment. These findings suggest that ASCs may differentiate into vascular endothelial cells or exert a paracrine effect in the peri-implant tissues, thereby relieving hypoxia, inflammation, and fibrosis, all of which are hallmarks of capsular contracture. In addition, our data suggest that ASC supplementation can help to prevent capsular contracture after implant-based breast reconstruction.

Several studies have shown that ASCs can effectively reduce fibrosis in various tissues [16–18]. As a result, ASCs are being widely studied as an alternative to treat fibrosis-related diseases in various organs. In animal models, it has been reported that intravenously-injected ASCs migrate to the liver and differentiate into hepatocytes, reducing hepatic fibrosis [18]. In addition, N-cadherin-transfected ASCs promote angiogenesis and cardiomyocyte differentiation, thereby reducing ventricular fibrosis and increasing the ejection fraction in a mouse model of ischemic heart disease [38]. Anti-fibrotic effects of ASCs have been repeatedly reported in the lungs, kidneys, and skin [17, 18, 39]. Based on these studies, inhibition of the TGFβ/Smad signaling axis, paracrine effects by various growth factors released from ASCs, anti-oxidative effects, and anti-inflammatory effects have been suggested as mechanisms by which ASCs suppress fibrosis [39]. In this study, we found that the mRNA levels of TGFβ, a master regulator of fibrosis that governs the production of extracellular matrix, and molecules operating downstream of TGFβ, were decreased in ASC-treated peri-implant capsules. In addition, macrophage infiltration was significantly reduced upon ASC treatment, which may also contribute to lowering myofibroblast activation [40]. Therefore, ASCs may reduce capsular fibrosis in the peri-implant tissue by inhibiting TGFβ signaling and inflammation, as has been observed in other tissues. Further investigation may be required to determine how these molecular alterations occur at the cellular level in the peri-implant capsular fibrosis model.

Hypoxia is another microenvironmental factor that regulates wound healing and tissue fibrosis [41, 42]. Fibrotic tissues are characterized by reduced capillary density, resulting in tissue hypoxia and thus stabilization of the HIF-1α transcription factor, which leads to the activation of fibrotic genes [41, 42]. Therefore, we also investigated the role of ASCs in modulating hypoxia. We confirmed that the tissue surrounding the implant where ASCs are distributed is less hypoxic, and HIF-1α is downregulated in this region. Considering that capillary density within the fibrous capsule was increased by ASC treatment, we believe that reduction of ischemia by enhanced angiogenesis may improve tissue hypoxia. We found that ASCs differentiate into vascular endothelial cells within the capsular tissue, which may contribute to increased vascularity. A recent study reported that cell-conditioned media from human embryonic stem cell-derived endothelial precursors reduced the capsular thickness around the implant by enhancing angiogenesis [43]. Therefore, ASCs may induce angiogenesis by their differentiation into vascular endothelial cells or through the paracrine effect of growth factors secreted by ASCs, both of which may help to relieve tissue hypoxia and fibrosis. Future studies should further examine the paracrine effect of ASCs by investigating the levels of various growth factors within the tissue surrounding the implant in response to ASC treatment.

One of the strengths of our study is that, unlike previous studies, we were able to track the injected ASCs by their GFP fluorescence, thereby confirming their survival, distribution, and differentiation. Using this method, we were able to demonstrate that injected ASCs were viable, distributed in the capsular tissue adjacent to the implant, and differentiated into vascular endothelial cells. These findings provide evidence to support that the reduction of capsular thickness and fibrosis were associated with injected ASCs. Similar to our study, previous studies have traced the fate of the fluorescent ASCs when they were co-injected at the time of fat grafting [19, 20]. These studies also showed that ASCs were differentiated into vascular endothelial cells [19, 20]. Considering that fat grafting also induces hypoxia, their results are consistent with our findings. Likewise, fluorescence-labeled ASCs may be further utilized to validate the effect of ASCs and their underlying mechanisms in various disease models in the future.

## Conclusions

We examined the role of ASCs in silicone implant-induced capsular fibrosis to study the capsular contracture that often occurs after implant-based breast reconstruction. Implanted ASCs were viable and transdifferentiated into vascular endothelial cells in peri-implant fibrous tissues. ASC treatment relieved capsular fibrosis around the silicone implant and lowered the levels of hypoxia and inflammation. Based on these findings, ASCs may be utilized as an adjuvant treatment to prevent capsular contracture after breast implantation.

## Data Availability

The data generated and/or analyzed during this study are available from the corresponding authors on reasonable request.

## Abbreviations

TGFβ: Transforming growth factor β
ASCs: Adipose-derived stem cells
GFP: Green fluorescent protein
B6: C57BL/6
PBS: Phosphate-buffered saline
H&E: Hematoxylin and eosin
αSMA: α-Smooth muscle actin
iNOS: Inducible nitric oxide synthase
qRT-PCR: Quantitative real-time polymerase chain reaction
Hif1α: Hypoxia-inducible factor 1α
Il-1β: Interleukin 1β
Il-6: Interleukin 6
Fn: Fibronectin
Col1α1: Collagen type I alpha 1
Col3α1: Collagen type III alpha 1
GADPH: Glyceraldehyde 3-phosphate dehydrogenase
M: Mean
SD: Standard deviation
